# Comparative Study on Hyperelastic Constitutive Models for the Static and Dynamic Behavior of Resilient Mounts

**DOI:** 10.3390/ma18020311

**Published:** 2025-01-11

**Authors:** Sung-Ju Park

**Affiliations:** 1 School of Electrical & Control Engineering, Tongmyong University, Busan 48520, Republic of Korea; parksj0314@tu.ac.kr; Tel.: +82-51-629-1657; 2 Open Grid Laboratory, Tongmyong University, Busan 48520, Republic of Korea

**Keywords:** resilient mount, hyperelastic constitutive models, force transmissibility, static and dynamic analysis

## Abstract

Resilient mounts play a vital role in anti-vibration and shock-absorption systems, making precise estimation of their static and dynamic stiffness essential for ensuring optimal mechanical performance and effective design. This study investigates the behavior of resilient mounts by analyzing their static and dynamic stiffness characteristics through the application of various hyperelastic constitutive models. Seven hyperelastic models were reviewed and systematically compared using numerical simulations, experimental data, and analytical solutions. The model parameters were calibrated and optimized based on experimental results obtained from quasi-static loading tests, including tension, compression, and shear conditions. Additionally, frequency response simulations were employed to evaluate the dynamic behavior of the resilient mounts under varying preload scenarios. The results provide practical insights into the performance of hyperelastic models and offer a comprehensive guideline for selecting suitable models for resilient mount applications, contributing to improved accuracy in both static and dynamic performance predictions.

## 1. Introduction

Resilient mounts are critical components in mechanical systems, serving as essential interfaces that mitigate vibrations and protect sensitive equipment from mechanical stress. These versatile devices play a pivotal role across diverse industries, including industrial machinery, shipbuilding, automotive engineering, and aerospace applications. By effectively absorbing and dissipating energy, resilient mounts enhance system stability, reduce noise, and prevent potential damage to connected structures and equipment. The performance of resilient mounts fundamentally depends on their ability to accurately predict and control static and dynamic stiffness characteristics. However, characterizing the mechanical behavior of these complex components presents significant challenges due to the nonlinear and highly sensitive nature of rubber-like materials. Researchers have developed various approaches to address these challenges, each contributing unique insights into material modeling and performance prediction.

Research on hyperelastic material modeling has evolved significantly. Amin et al. [1] made a fundamental contribution by developing an improved hyperelasticity model for natural and high damping rubbers, introducing a parameter identification scheme that captures the Fletcher–Gent effect under compression and shear conditions. Building on this foundation, Sasso et al. [2] advanced the field by developing an optical method for characterizing hyperelastic materials, demonstrating the effectiveness of Mooney–Rivlin and Ogden models across different loading conditions. Li and Wei [3] conducted extensive testing to validate classic strain energy functions. Gorash et al. [4] developed CAE-based methods for model parameter identification. Nörenberg and Mahnken [5] developed an innovative parameter identification framework for rubber materials by introducing artificial spatially distributed test data. Wu et al. [6] developed a hybrid optimization method combining pattern search and Levenberg–Marquardt algorithms to fit hyperelastic material models to multi-axial experimental data. Dynamic characterization methods have seen substantial development. Kari [7] introduced an indirect measurement technique for determining blocked dynamic transfer stiffness in the audible frequency range, significantly improving measurement accuracy. Lee et al. [8] further advanced this area by developing an efficient method to predict dynamic stiffness under preload conditions using the dynamic ratio concept. Moro and Biot [9] specifically addressed marine applications by developing methods for characterizing dynamic transfer stiffness of large resilient mounts, while Carrella [10] contributed techniques for identifying nonlinear characteristics using transmissibility measurements. Külcü [11] proposed new constitutive models specifically designed for rubber-like materials. Plagge et al. [12] introduced methods accounting for stress-induced microstructural changes. This work was complemented by Miroshnychenko and Green [13] who developed heuristic search methods for strain-energy functions. Numerical modeling approaches have been systematically developed to improve the predictive capabilities of mechanical systems. Nicholson et al. [14] highlighted the computational challenges in finite element analysis of hyperelastic components, particularly regarding large deformations and material near-incompressibility. Seibert and Schöcke [15] conducted direct comparisons of recent models, while Horgan and Smayda [16] emphasized the importance of including second strain invariants in constitutive modeling. These experimental studies have been essential for understanding model limitations and applicability ranges. Luo et al. [17] introduced innovative approaches combining energy dissipation methods for quasi-static loading with Natural Frequency Region analysis for dynamic impact. Fragasso et al. [18] developed a methodology optimizing Yeoh model coefficients through finite element simulation, while Íñiguez-Macedo et al. [19] demonstrated the superiority of the Mooney–Rivlin model through multi-response surface optimization. Fragasso and Moro [20] developed an experimental procedure to characterize the dynamic properties of marine diesel engine resilient mounts. Environmental effects and material behavior have received increasing attention. Gong et al. [21] investigated temperature and speed effects on rubber dynamic stiffness in railway applications, revealing significant impacts on damping performance. Emminger et al. [22] advanced the understanding of thermoplastic materials through comprehensive characterization and validation. Liu et al. [23] and Danko et al. [24] contributed to understanding dynamic characteristics through nonlinear mechanical modeling and viscoelastic material behavior analysis. Recent developments have focused on model refinement and validation. He et al. [25] conducted a comprehensive review of 85 hyperelastic models, providing valuable insights into model selection for different rubber types. Sun et al. [26] improved Mooney–Rivlin parameter identification under preload conditions, while Mollaee et al. [27] developed innovative optical-based techniques for parameter identification using inhomogeneous deformations.

This study undertakes a comprehensive numerical review of seven isotropic hyperelastic constitutive models and their application to resilient mount behavior. Through systematic evaluation of model performance under both static and dynamic conditions, we establish clear guidelines for model selection in specific applications. Our research advances the current understanding by conducting extensive analysis of model accuracy across multiple deformation modes and evaluating dynamic performance through detailed modal analysis. The investigation provides practical guidelines for model selection while identifying key challenges and future research directions in the field. These findings contribute significant insights for improving resilient mount design and analysis, particularly in applications where precise vibration control and long-term reliability are crucial. The results of this study offer valuable guidance for engineers and researchers working on vibration isolation systems across various industrial applications.

## 2. Theoretical Background

Hyperelastic materials are characterized by their ability to undergo large deformations and return to their original shape upon unloading, exhibiting nonlinear stress–strain behavior. The constitutive models describing these materials are derived from strain energy density functions, which relate the strain energy stored in the material to the deformation state. These models are particularly important for describing rubber-like materials where the stress–strain relationship is nonlinear and path-independent.

### 2.1. Strain Energy Density Function

Based on continuum mechanics, isotropic hyperelastic materials for rubberlike materials are described using an elastic strain energy density function, W, which is defined in terms of the strain invariants of the right Cauchy–Green deformation tensor $\left(I_{1},I_{2},I_{3}\right)$ or the principal stretches $\left(\lambda_{1},\;\lambda_{2},\lambda_{3}\right).$ The three principal strain invariants are as follows:

(1) $$I_{1}=\lambda_{1}^{2}+\lambda_{2}^{2}+\lambda_{3}^{2},$$

(2) $$I_{2}=\lambda_{1}^{2}\lambda_{2}^{2}+\lambda_{2}^{2}\lambda_{3}^{2}+\lambda_{1}^{2}\lambda_{3}^{2},$$

(3) $$I_{3}=\lambda_{1}^{2}\lambda_{2}^{2}\lambda_{3}^{2}.$$

The strain energy density function can be divided into the deviatoric part, $W_{Dev}$, which accounts for shape changes, and the volumetric part, $W_{Vol}$, which accounts for volume changes:

(4) $$W=W_{Dev}\left({\bar{I}}_{1},{\bar{I}}_{2}\right)+W_{Vol}\left(\bar{J}\right),$$

where ${\bar{I}}_{1}=I_{1}/J^{\frac{2}{3}},{\bar{I}}_{2}=I_{2}/J^{4/3}$. $J=\sqrt{I_{3}}$ is the elastic volume ratio.

For materials that are homogeneous, isotropic, and incompressible (or nearly incompressible), such as rubber, the strain energy density function simplifies. Incompressibility implies that the third strain invariant $I_{3}$, which is related to volume changes, remains equal to one. Therefore, the strain energy density can be expressed using only the deviatoric components associated with shape changes, denoted as $W_{dev}$, as shown in Equation (5):

(5) $$W=W_{Dev}\left(I_{1},\;I_{2}\right).$$

Numerous strain energy potentials have been developed to characterize incompressible isotropic elastomers, each tailored to capture specific material behaviors. This study examines these constitutive models on deviatoric strain energy function, comparing their characteristics for both static and dynamic applications in finite element analysis (FEA) contexts. Through this comparative approach, the paper aims to evaluate the fidelity and computational efficiency of each model when applied to resilient mount behavior.

### 2.2. Hyperelastic Model

The selected hyperelastic models in this study represent diverse modeling approaches: Arruda–Boyce, Marlow, Mooney–Rivlin, Neo-Hookean, Ogden, polynomial, reduced polynomial, Yeoh, Valanis–Landel, and Van der Waals models. Table 1 summarizes the strain energy density function for incompressible rubberlike materials. Each model offers unique insights and limitations, making comprehensive comparative analysis crucial for understanding material behavior.

#### 2.2.1. Arruda–Boyce Model

Arruda and Boyce [28] present a hyperelastic constitutive model based on the behavior of molecular chain networks. The Arruda–Boyce model assumes that the total strain energy is the sum of the strain energy contributions from individual molecular chains. The alternative form of the Arruda–Boyce model is described as:

(6) $$W=\mu \sum_{i=1}^{5}\frac{C_{i}}{\lambda_{m}^{2i-2}}\left({\bar{I}}_{1}^{i}-3^{i}\right),$$

$$C_{1}=\frac{1}{2},\;C_{2}=\frac{1}{20},\;C_{3}=\frac{11}{1050},\;C_{4}=\frac{19}{7000},\;C_{5}=\frac{519}{673,750}\;.$$

μ and $\lambda_{m}$ are the initial shear modulus and the stretch at which the polymer network becomes locked.

#### 2.2.2. Marlow Model

The Marlow hyperelastic model [29] is notable for its simplicity, as it eliminates the need for extensive parameter fitting by directly defining the strain energy function based on experimental test data. Consequently, the strain energy density function depends solely on the first invariant, making the second invariant irrelevant. This approach requires only one experimental test to characterize the material’s behavior. The Marlow model is particularly effective for predicting material behavior under specific deformation modes, making it an excellent initial choice when test data are limited. By minimizing parameterization, the model provides reliable results and enables efficient material representation with minimal input.

#### 2.2.3. Mooney–Rivlin Model

Mooney–Rivlin model [30,31] is one of the most widely used hyperelastic material models for analyzing rubber components. It provides a more flexible framework compared to simpler models, as it incorporates both the first and second strain invariants of the deformation tensor. This enables the Mooney–Rivlin model to capture a broader range of deformation behaviors in rubber-like materials. The strain energy density function is defined as:

(7) $$W=C_{10}\left(I_{1}-3\right)+C_{10}\left(I_{2}-3\right).$$

The Mooney–Rivlin model is particularly effective for describing the mechanical behavior of rubber under moderate to large deformations. By adjusting the coefficients $C_{10}$ and $C_{01}$, the model can fit a wide range of experimental stress–strain data, making it a versatile choice in the analysis of elastomeric materials.

#### 2.2.4. Neo-Hookean Model

The Neo-Hookean model is a specific and simplified case of the Mooney–Rivlin model. It assumes that the material response depends solely on the first strain invariant, $I_{1}$, making it a straightforward model for describing the nonlinear elastic behavior of incompressible rubber-like materials. The strain energy density function in this formulation is expressed as:

(8) $$W=C_{10}\left(I_{1}-3\right),$$

where $C_{10}$ is a material constant. The Neo-Hookean model is commonly used for moderate deformations and serves as a foundational model in hyperelastic material modeling. While it is less accurate for capturing highly nonlinear behaviors compared to more complex models, it is computationally efficient and provides a good approximation for small to moderate deformations.

#### 2.2.5. Ogden Model

Ogden [32] model is an extension of the Mooney–Rivlin model, designed to describe the nonlinear elastic behavior of rubber-like materials. Unlike models based solely on strain invariants, the Ogden model is expressed directly in terms of the three principal stretches $\left(\lambda_{1},\lambda_{2},\lambda_{3}\right)$. It incorporates a series expansion involving material constants $\mu_{i}$ and $\alpha_{i}$, providing a highly flexible framework to capture complex material responses. For an incompressible material, the strain energy density function is given as:

(9) $$W=\sum_{i=1}^{N}\frac{2\mu_{i}}{\alpha_{i}^{2}}\left(\lambda_{1}^{\alpha_{i}}+\lambda_{2}^{\alpha_{i}}+\lambda_{1}^{-\alpha_{i}}\lambda_{2}^{-\alpha_{i}}-3\right).$$

This model is particularly effective for capturing large deformations in elastomers and soft tissues. By fitting multiple terms $\left(N\right)$ in the series, the Ogden model can represent a wide range of stress–strain behaviors with high accuracy, making it a versatile choice for complex hyperelastic materials.

#### 2.2.6. Yeoh Model

The Yeoh model [33,34] is based on the first invariant, and the strain density function for an incompressible material can be expressed by setting i=1, 2, 3 and j=0 in the polynomial form. This simplifies the expression to:

(10) $$W=\sum_{i=1}^{3}C_{i0}{\left(I_{1}-3\right)}^{i},$$

(11) $$W=C_{10}{\left(I_{1}-3\right)}^{2}+C_{20}{\left(I_{1}-3\right)}^{2}+C_{30}{\left(I_{1}-3\right)}^{3}.$$

The initial shear modulus and bulk modulus are given by: $\mu_{0}=2C_{10}$ and $K_{0}=\frac{2}{D_{1}}$.

#### 2.2.7. Van Der Waals Model

The Van der Waals model [35] is grounded in thermodynamic principles, providing a more physically realistic interpretation of material behavior. This model is particularly well-suited for materials where significant interatomic or intermolecular forces dominate, as it explicitly incorporates these interactions into the strain energy density function. By accounting for these forces, the Van der Waals model delivers an enhanced representation of material responses under large deformations, making it a valuable tool for predicting the mechanical behavior of elastomers and polymers. Its thermodynamic foundation ensures consistency with the physical laws governing material behavior, enhancing its reliability and accuracy in practical applications. The strain energy density function for the Van der Waals model is given as follows:

(12) $$W=\mu \left\{-\left(\lambda_{m}^{2}-3\right)\left[\ln\left(1-\theta \right)+\theta \right]-\frac{2}{3}\alpha {\left(\frac{\tilde{I}-3}{2}\right)}^{\frac{3}{2}}\right\},$$

where

(13) $$\tilde{I}=\left(1-\beta \right){\bar{I}}_{1}+\beta {\bar{I}}_{2,}$$

(14) $$\theta =\sqrt{\frac{\tilde{I}-3}{\lambda_{m}^{2}-3}}.$$

## 3. Hyperelastic Model Parameters Identification

### 3.1. Previous Experiments for Material Constant Calibration

**Figure 1:**
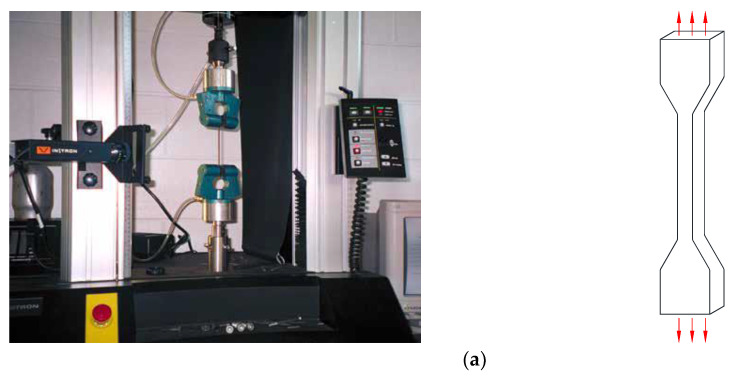

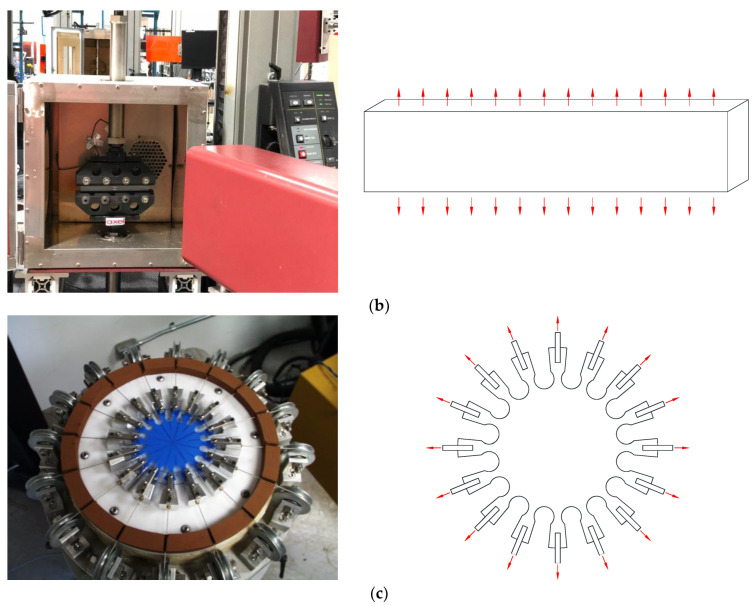
The schematic photo (source: https://www.axelproducts.com/ (accessed on 26 December 2024)) and illustrations of deformation modes [36] (**a**) uniaxial tension, (**b**) planar tension, and (**c**) equi-biaxial tension.

**Figure 2:**
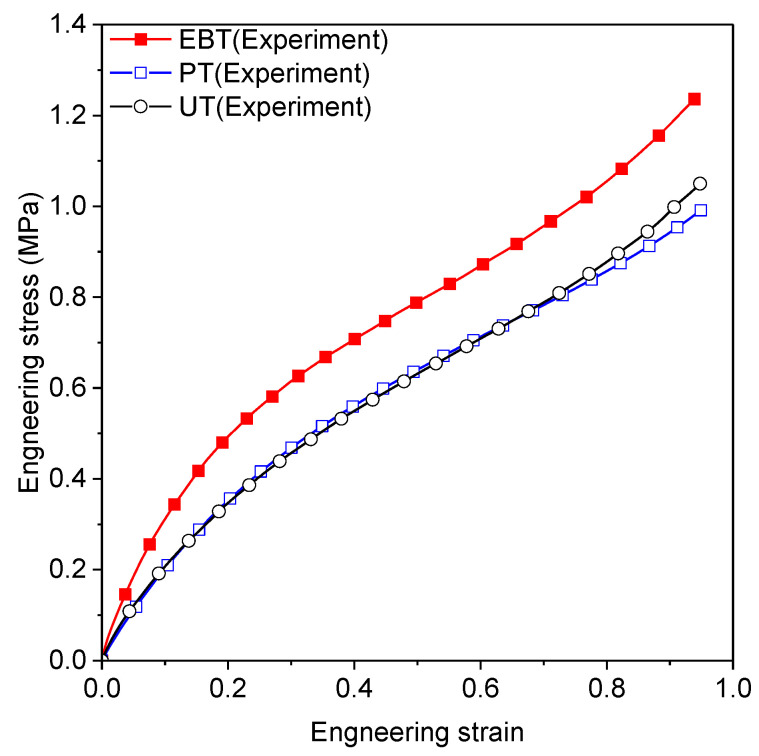
Experimental results for uniaxial tension, planar tension, and equi-biaxial tension test.

In this section, standard tests from a previous study [36] are introduced to calibrate the parameters of each hyperelastic model. Uniaxial tension (UT), planar tension (PT), and equi-biaxial tension (EBT) were conducted to represent distinct deformation modes, all performed at quasi-static speeds and room temperature. A schematic illustration of each deformation mode is shown in Figure 1. Each test was applied for five loading cycles at stretch levels of 30%, 50%, 70%, and 100% to mitigate the Mullins effect in the rubber material. Figure 2 shows the engineering stress–strain curves obtained from the final loading cycle.

### 3.2. Review of Hyperelastic Models

An optimization using objective function $f\left(Q_{n}\right)$ was conducted to calibrate hyperelastic model parameters. The optimization algorithm was implemented within the nonlinear least-squares minimization problem,

(15) $$f\left(Q_{n}\right)={\arg min}_{q}\left(\frac{1}{2}\sum_{n=1}^{3}Q_{n}\right),$$

with

(16) $$Q_{n}=\frac{1}{2}\sum_{m=1}^{M_{n}}{\left(P_{n}-P_{nm}\right)}^{2},$$

**Figure 3:**
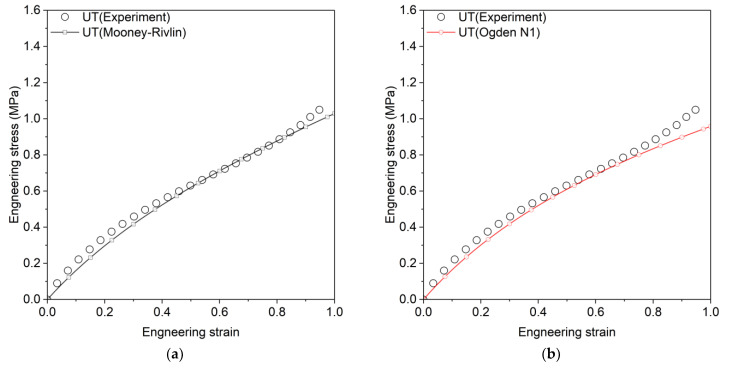

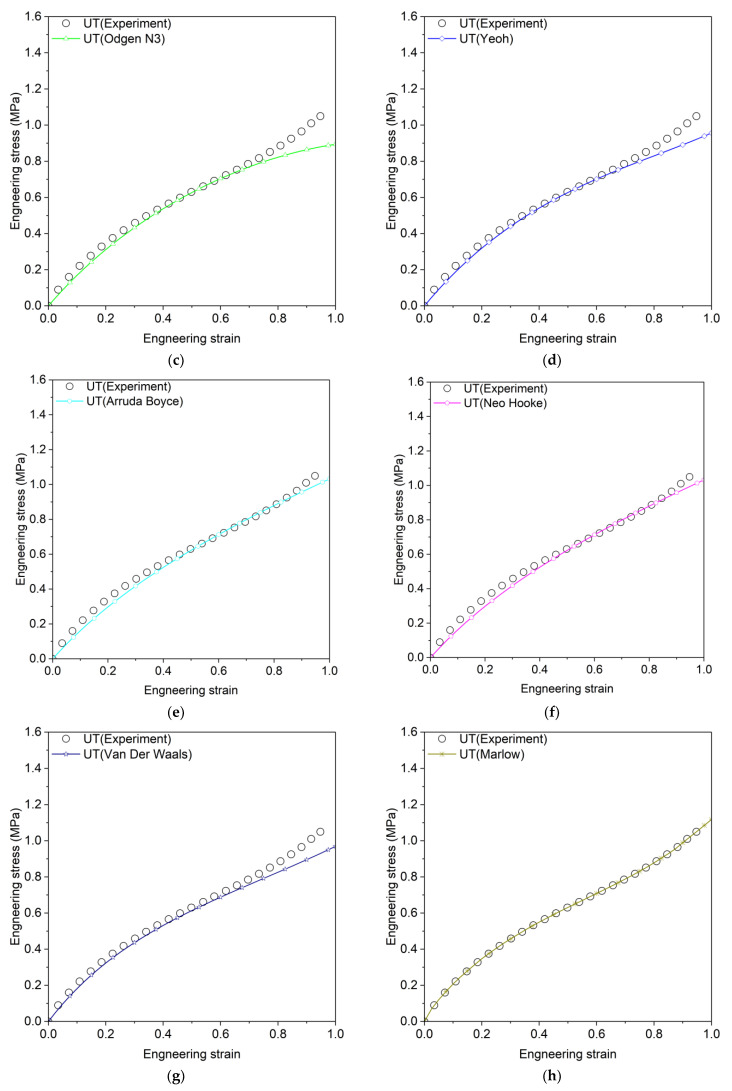
Engineering stress–strain curves for uniaxial tension, comparing experimental results with predictions from various hyperelastic models, (**a**) Mooney–Rivlin, (**b**) Ogden N1, (**c**) Ogden N3, (**d**) Yeoh, (**e**) Arruda–Boyce, (**f**) Neo-Hookean, (**g**) Van Der Waals, (**h**) Marlow.

**Figure 4:**
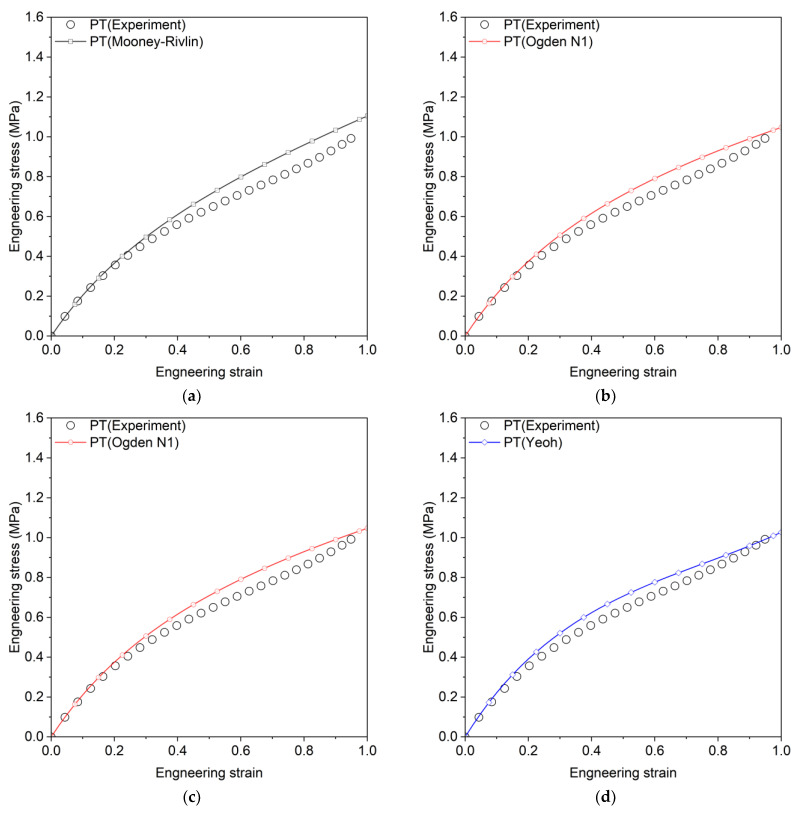

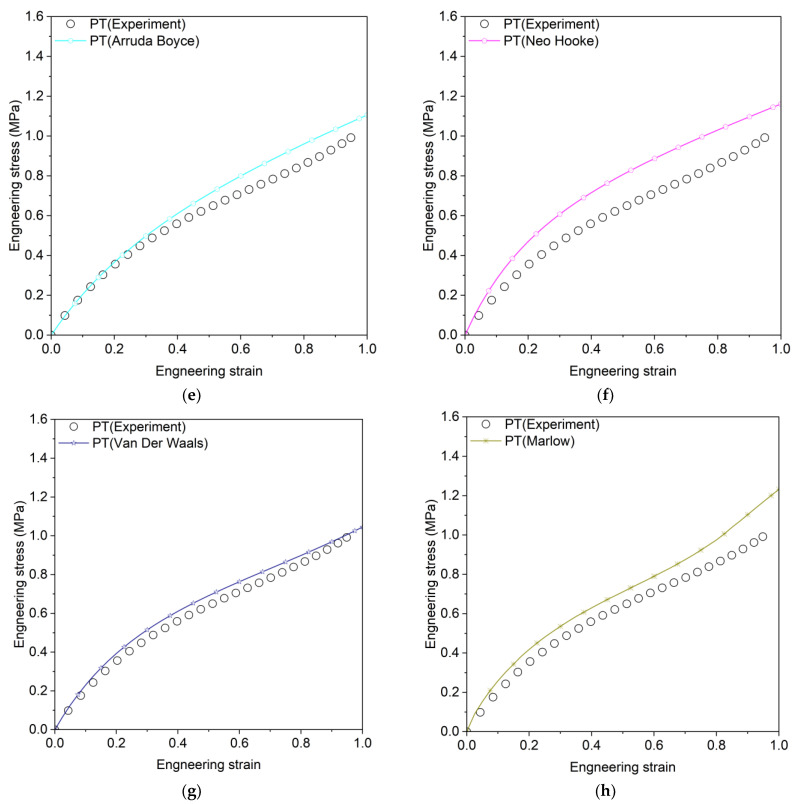
Engineering stress–strain curves for planar tension, comparing experimental results with predictions from various hyperelastic models, (**a**) Mooney–Rivlin, (**b**) Ogden N1, (**c**) Ogden N3, (**d**) Yeoh, (**e**) Arruda–Boyce, (**f**) Neo-Hookean, (**g**) Van Der Waals, (**h**) Marlow.

**Figure 5:**
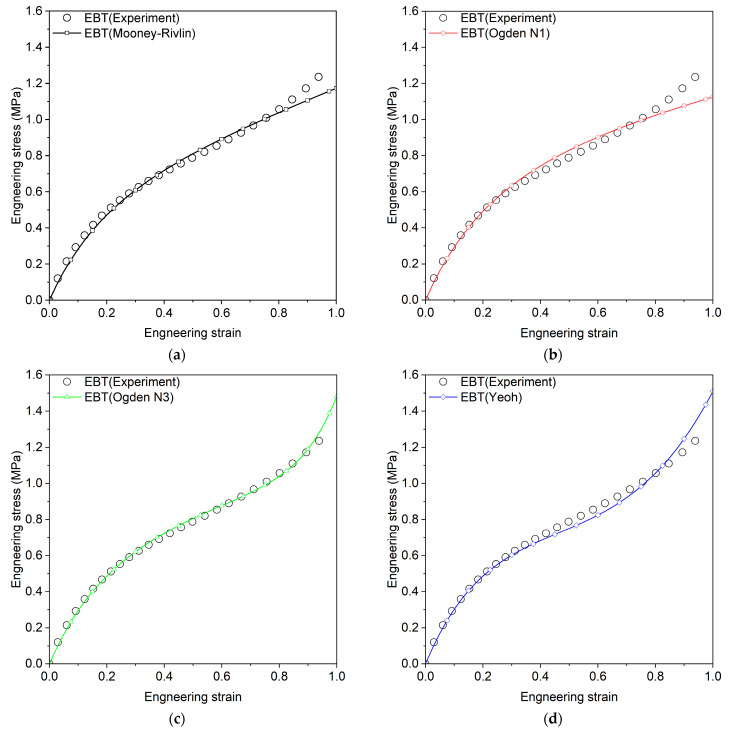

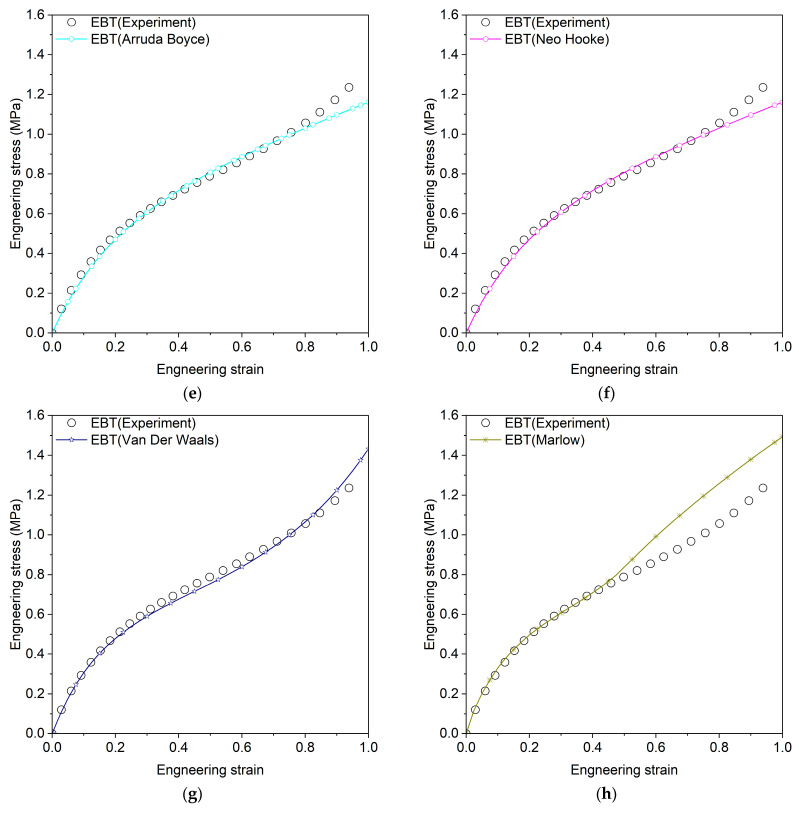
Engineering stress–strain curves for equi-biaxial tension, comparing experimental results with predictions from various hyperelastic models, (**a**) Mooney–Rivlin, (**b**) Ogden N1, (**c**) Ogden N3, (**d**) Yeoh, (**e**) Arruda–Boyce, (**f**) Neo-Hookean, (**g**) Van Der Waals, (**h**) Marlow.

Here, q represents the material parameters to be estimated, and n indexes three calibration datasets (UT, PT, and EBT). $P_{n}$ represents the model prediction for experimental parameter $P_{nm}$, where m is the data point index and $M_{n}$ is the total number of data points per experiment. The Marlow model was calibrated using only uniaxial tension data. Table 2 presents the calibrated parameters and coefficient of determination ($R^{2}$) for each model, where $R^{2}$ values range from 0 to 1, with 1 indicating a perfect fit. The Mooney–Rivlin, Van Der Waals, and Yeoh models show consistently high $R^{2}$ values across all tests, while the Marlow model achieves perfect fit only for UT but performs poorly in other tests. Figure 3, Figure 4 and Figure 5 compare experimental and predicted engineering stress–strain curves, showing that Ogden N3 and Neo-Hookean models excel in EBT but underperform in PT tests.

## 4. Finite Element Implementation of Resilient Mount

The finite element analysis (FEA) of the resilient mount was conducted to investigate its static and dynamic characteristics across various hyperelastic models. This resilient mount consists of three components (a flange, center plate, and rubber element) designed for vibration isolation in compliance with the MIL-M-17508F [37] standard. Static and dynamic simulations were performed using the commercial FEA software Abaqus 2023.

The material models were implemented using custom material definitions based on our experimental data rather than using pre-existing material libraries. For the steel parts, Young’s modulus, density, and Poisson’s ratio were set to 210,000 MPa, 7850 kg/m^3^, and 0.3, respectively. For the rubber component, we created new material definitions for each hyperelastic model in Abaqus, inputting the calibrated parameters obtained from our optimization process (shown in Table 2). These parameters were derived from experimental results of quasi-static tests (detailed in Section 3.1). This approach ensures that the material behavior in our simulations accurately reflects our specific experimental conditions rather than relying on generic material properties.

**Figure 6:**
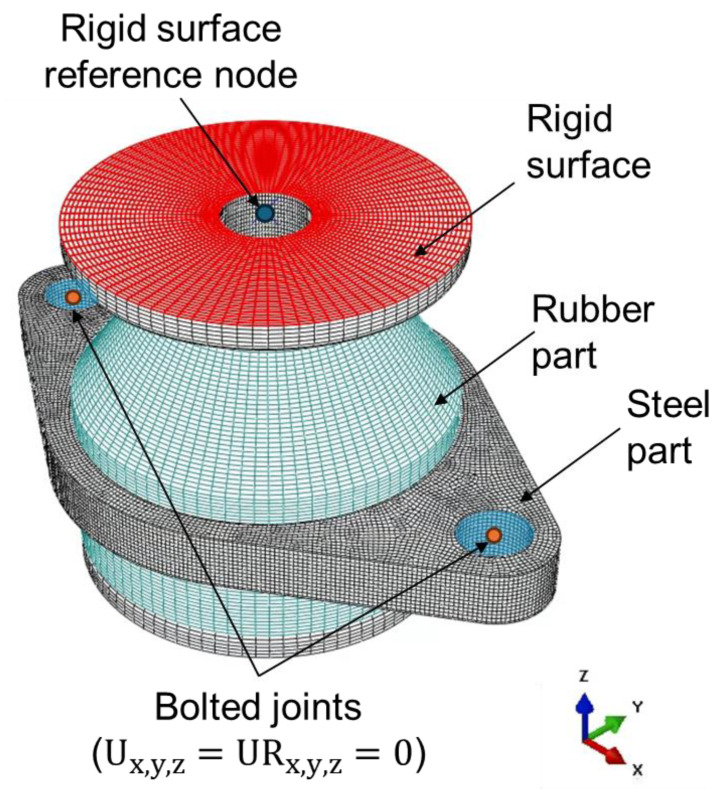
Finite element model of resilient mount [36].

The finite element analysis was carried out for compression deformation mode of resilient mount. FE models were created using reduced integration elements as shown in Figure 6. Specifically, the rubber parts utilized hybrid elements, which are provided by Abaqus for compressible properties. The mesh size was determined through convergence tests. A parametric study was conducted to evaluate the influence of different hyperelastic models on the rubber material’s behavior.

### 4.1. Static Analysis

**Figure 7:**
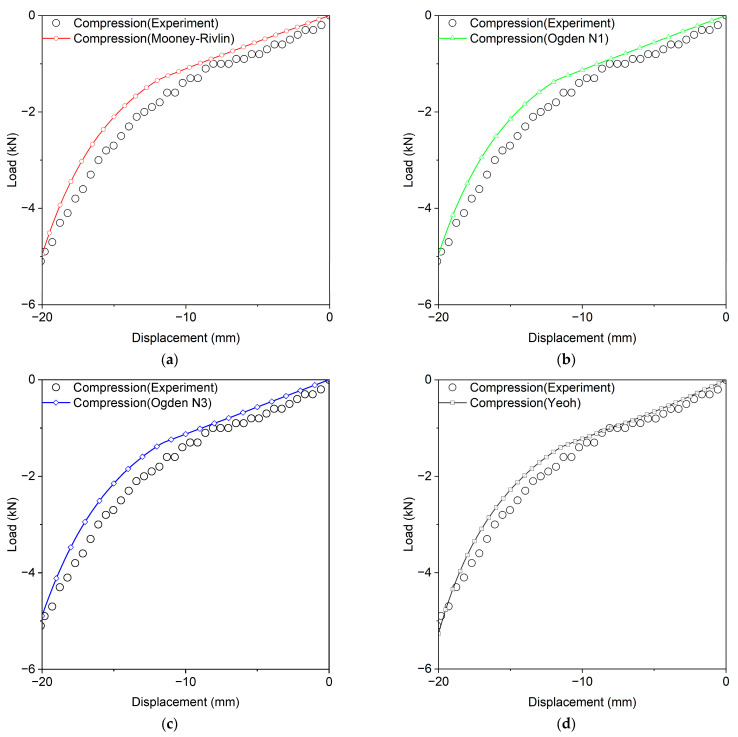

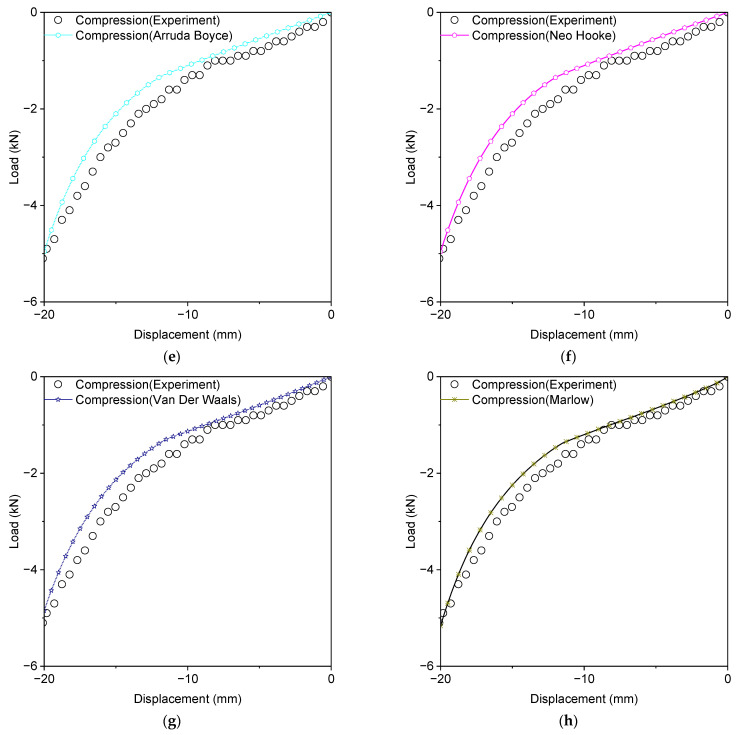
Comparison of force–displacement response between experimental and numerical results for compression test, (**a**) Mooney–Rivlin, (**b**) Ogden N1, (**c**) Ogden N3, (**d**) Yeoh, (**e**) Arruda–Boyce, (**f**) Neo-Hookean, (**g**) Van Der Waals, (**h**) Marlow.

Figure 7 illustrates the stroke displacement and reaction force curves, comparing experimental data with numerical results. Table 3 presents the strain energy ($U=\int_{0}^{u}Fdu$) for each hyperelastic model. The Yeoh model demonstrates superior performance, with the lowest error of 6.92%, suggesting that its formulation is particularly well-suited to capturing the strain energy behavior of this material. In contrast, the Mooney–Rivlin and Neo-Hookean models exhibit higher errors of 14.83% and 12.19%, respectively. The wide variation in prediction accuracy, with errors ranging from 6.92% to 14.83%, underscores the critical role of model selection in the characterization of hyperelastic materials. This comparative analysis highlights the importance of carefully evaluating and selecting an appropriate constitutive model to accurately predict the mechanical behavior of hyperelastic materials.

### 4.2. Dynamic Analysis

**Figure 8:**
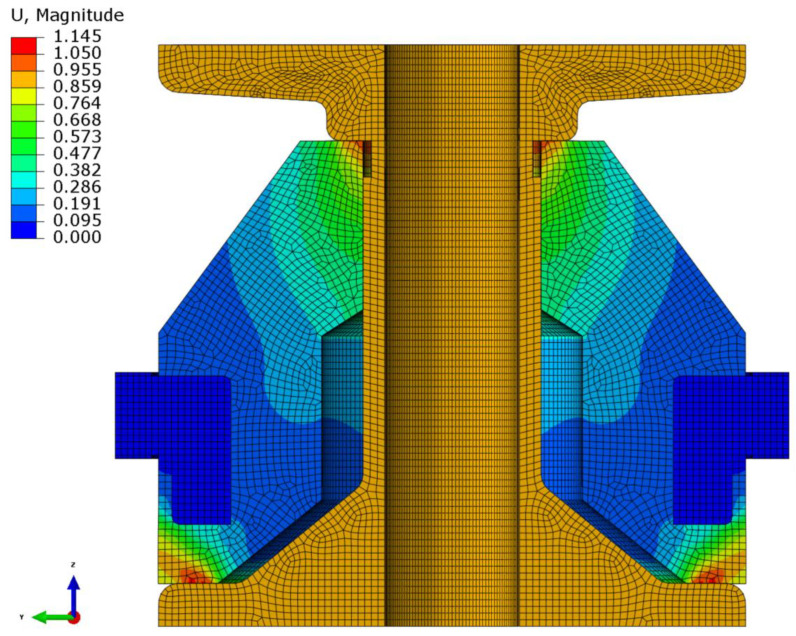
Modal analysis: first mode cross-section.

Modal analyses were conducted to evaluate the dynamic characteristics of the resilient mount. Figure 8 illustrates the first mode shape obtained from modal analysis. The frequencies across four modes for different hyperelastic models are presented in Table 4. The resonant frequency of the resilient mount was experimentally determined to be 114 Hz using force transmissibility [36]. The dynamic analysis reveals distinct patterns in model performance. The Yeoh model shows the closest prediction to experimental results with a first mode frequency of 113.61 Hz (0.34% deviation), followed by Ogden N3 (111.67 Hz, 2.04% deviation) and Odden N1 (111.54 Hz, 2.16% deviation). The Van Der Waals model consistently predicts higher frequencies across all modes, with its first mode prediction (118.44 Hz) showing the largest deviation of 3.89% from experimental data. In contrast, the Arruda–Boyce, Marlow, Mooney–Rivlin, and Neo-Hookean models predict lower frequencies, clustering around 108.94–108.98 Hz (approximately 4.4% deviation). Analysis of higher modes reveals important trends in model robustness. The relative deviation between models increases with higher modes, ranging from approximately 10 Hz difference in first mode predictions to nearly 30 Hz in fourth mode predictions. This growing disparity suggests that model selection becomes increasingly critical when analyzing higher-frequency applications. The Yeoh model maintains consistent performance across all modes, showing balanced prediction capability for both lower and higher frequencies. The correlation between static and dynamic performance is particularly noteworthy. Models showing better strain energy prediction in static analysis (as discussed in Section 4.1) generally demonstrate more accurate frequency predictions. This correlation validates the importance of comprehensive material characterization in both static and dynamic domains for accurate resilient mount design. These findings provide practical guidelines for model selection in vibration isolation applications, particularly when both static and dynamic performance are critical design considerations. This comprehensive analysis establishes the Yeoh model’s superior capability in predicting both static and dynamic behavior of resilient mounts, making it particularly suitable for applications requiring accurate vibration prediction across multiple frequency ranges.

## 5. Conclusions

This study’s comprehensive analysis of hyperelastic constitutive models for resilient mounts yielded several key findings. The Yeoh model achieved superior accuracy with 6.92% deviation in strain energy predictions and 0.34% deviation in natural frequency predictions (113.61 Hz vs. experimental 114 Hz). Other models showed significantly larger deviations: Van Der Waals (13.47%), Mooney–Rivlin (14.81%), and Arruda–Boyce (14.78%) in strain energy predictions. Dynamic analysis revealed frequency prediction variations of up to 8.7% across models (108.94–118.44 Hz).

For applications requiring both static and dynamic accuracy, the Yeoh model provides optimal performance, while the Marlow model is suitable for uniaxial-dominated applications but shows limitations in multi-axial loading. The Neo-Hookean model offers computational efficiency but sacrifices accuracy in complex loading conditions. Multi-axial test data integration proved essential for accurate parameter calibration, and hybrid element formulation improved numerical stability in compression simulations. Model selection significantly impacts computational cost versus accuracy trade-offs.

Several challenges remain for future research. These include improving model accuracy at large deformations (>100% strain), reducing computational cost for complex geometry applications, and developing standardized testing protocols for model calibration. Future work should focus on developing adaptive modeling approaches for varying environmental conditions, investigating temperature and frequency dependencies in hyperelastic parameters, and integrating machine learning techniques for parameter optimization. Additionally, extending model validation to cyclic loading and fatigue conditions and developing simplified calibration procedures for industrial applications would enhance the practical implementation of these models.

The findings from this study establish a robust framework for selecting appropriate hyperelastic models in resilient mount applications, particularly in scenarios requiring accurate prediction of both static and dynamic behavior. The methodology presented provides a systematic approach for evaluating and implementing hyperelastic models in vibration isolation applications.

## Figures and Tables

**Table 1:** Summary of the strain energy density function.

Model	Strain Energy Density Function	Parameters
Arruda–Boyce	$W=\mu \sum_{i=1}^{5}\frac{C_{i}}{\lambda_{m}^{2i-2}}\left({\bar{I}}_{1}^{i}-3^{i}\right)$	$\mu ,\;\lambda_{m}$
Marlow	$W=W_{dev}\left(I_{1}\right)$	-
Mooney–Rivlin	$W=C_{10}\left(I_{1}-3\right)+C_{01}\left(I_{2}-3\right)$	$C_{10},\;C_{01}$
Neo-Hookean	$W=C_{10}\left(I_{1}-3\right)$	$C_{10}$
Ogden	$W=\sum_{i=1}^{N}\frac{2\mu_{i}}{\alpha_{i}^{2}}\left(\lambda_{1}^{\alpha_{i}}+\lambda_{2}^{\alpha_{i}}+\lambda_{1}^{-\alpha_{i}}\lambda_{2}^{-\alpha_{i}}-3\right)$	$\mu_{i},\alpha_{i}$
Van Der Waals	$W=\mu \left\{-\left(\lambda_{m}^{2}-3\right)\left[\ln\left(1-\theta \right)+\theta \right]-\frac{2}{3}\alpha {\left(\frac{\tilde{I}-3}{2}\right)}^{\frac{3}{2}}\right\}$	$\mu ,\lambda_{m},\alpha ,\beta$
Yoeh	$W=C_{10}\left(I_{1}-3\right)+C_{20}{\left(I_{1}-3\right)}^{2}+C_{30}{\left(I_{1}-3\;\right)}^{3}$	$C_{10},C_{20},C_{30}$

**Table 2:** Calibrated hyperelastic model parameters.

Model	Parameters (Pa)	$R^{2}$		
		UT	EBT	PT
Arruda–Boyce	$\mu =589,757.637,\;\lambda_{m}=1,983,949,010$,	0.9879	0.9882	0.9309
Marlow	-	1.0	0.8525	0.8795
	$C_{10}=293,491.184,\;C_{01}=1095.312876$	0.9874	0.9899	0.9322
Neo-Hookean	$C_{10}=294,878.855$	0.9879	0.9882	0.6803
Odden N1	$\mu_{1}=607,775.779,\;\alpha_{1}=1,683,468.1$	0.973	0.9803	0.9539
Ogden N3	$\mu_{1}=2,767,873.68,\;\alpha_{1}=2,993,838.76,\;\mu_{2}=-2,148,600.07,$ $\lambda_{2}=3,276,269.1,\;\mu_{3}=22.2162626,\;\lambda_{3}=-8,764,509.87$	0.9643	0.9978	0.5970
Van Der Waals	$\mu =696,734.166,\;\lambda_{m}=4,470,344.47,$ α=727,629.666, β=0.0	0.9769	0.9926	0.9738
Yoeh	$C_{10}=376,965.272,\;C_{20}=-89,314.94317,$ $C_{30}=27,946.34328$	0.9773	0.9879	0.9663

**Table 3:** Comparison of strain energy between experimental data and numerical predictions using various hyperelastic models.

Label	Strain Energy (J)	Error (%)
Experiment	34.446	-
Arruda–Boyce	29.354	14.78256
Marlow	31.685	8.015444
Mooney–Rivlin	29.344	14.81159
Neo-Hookean	29.354	14.78256
Odden N1	29.843	13.36294
Ogden N3	29.847	13.35133
Van Der Waals	29.806	13.47036
Yoeh	32.061	6.923881

**Table 4:** Frequencies of the first four modes for different hyperelastic models.

Label	Order (Hz)			
	1	2	3	4
Arruda–Boyce	108.98	271.14	288.79	338.04
Marlow	108.94	271.03	288.66	337.91
Mooney–Rivlin	108.98	271.14	288.79	338.04
Neo-Hookean	108.98	271.14	288.79	338.04
Odden N1	111.54	277.81	295.89	346.24
Ogden N3	111.67	277.84	295.93	346.40
Van Der Waals	118.44	294.68	313.89	367.42
Yoeh	113.61	282.67	301.08	352.43

## Data Availability

The original contributions presented in the study are included in the article, further inquiries can be directed to the corresponding authors.
